# UPLC-MRM Mass Spectrometry Method for Measurement of the Coagulation Inhibitors Dabigatran and Rivaroxaban in Human Plasma and Its Comparison with Functional Assays

**DOI:** 10.1371/journal.pone.0145478

**Published:** 2015-12-23

**Authors:** Joachim Kuhn, Tatjana Gripp, Tobias Flieder, Marcus Dittrich, Doris Hendig, Jessica Busse, Cornelius Knabbe, Ingvild Birschmann

**Affiliations:** 1 Institute for Laboratory and Transfusion Medicine, Heart and Diabetes Center North Rhine-Westphalia, Ruhr University Bochum, Bad Oeynhausen, Germany; 2 Department of Bioinformatics, Biocenter, University of Würzburg, Würzburg, Germany; IIBB-CSIC-IDIBAPS, SPAIN

## Abstract

**Introduction:**

The fast, precise, and accurate measurement of the new generation of oral anticoagulants such as dabigatran and rivaroxaban in patients’ plasma my provide important information in different clinical circumstances such as in the case of suspicion of overdose, when patients switch from existing oral anticoagulant, in patients with hepatic or renal impairment, by concomitant use of interaction drugs, or to assess anticoagulant concentration in patients’ blood before major surgery.

**Methods:**

Here, we describe a quick and precise method to measure the coagulation inhibitors dabigatran and rivaroxaban using ultra-performance liquid chromatography electrospray ionization-tandem mass spectrometry in multiple reactions monitoring (MRM) mode (UPLC-MRM MS). Internal standards (ISs) were added to the sample and after protein precipitation; the sample was separated on a reverse phase column. After ionization of the analytes the ions were detected using electrospray ionization-tandem mass spectrometry. Run time was 2.5 minutes per injection. Ion suppression was characterized by means of post-column infusion.

**Results:**

The calibration curves of dabigatran and rivaroxaban were linear over the working range between 0.8 and 800 μg/L (r >0.99). Limits of detection (LOD) in the plasma matrix were 0.21 μg/L for dabigatran and 0.34 μg/L for rivaroxaban, and lower limits of quantification (LLOQ) in the plasma matrix were 0.46 μg/L for dabigatran and 0.54 μg/L for rivaroxaban. The intraassay coefficients of variation (CVs) for dabigatran and rivaroxaban were < 4% and 6%; respectively, the interassay CVs were < 6% for dabigatran and < 9% for rivaroxaban. Inaccuracy was < 5% for both substances. The mean recovery was 104.5% (range 83.8–113.0%) for dabigatran and 87.0% (range 73.6–105.4%) for rivaroxaban. No significant ion suppressions were detected at the elution times of dabigatran or rivaroxaban. Both coagulation inhibitors were stable in citrate plasma at -20°C, 4°C and even at RT for at least one week. A method comparison between our UPLC-MRM MS method, the commercially available automated Direct Thrombin Inhibitor assay (DTI assay) for dabigatran measurement from CoaChrom Diagnostica, as well as the automated anti-Xa assay for rivaroxaban measurement from Chromogenix both performed by ACL-TOP showed a high degree of correlation. However, UPLC-MRM MS measurement of dabigatran and rivaroxaban has a much better selectivity than classical functional assays measuring activities of various coagulation factors which are susceptible to interference by other coagulant drugs.

**Conclusions:**

Overall, we developed and validated a sensitive and specific UPLC-MRM MS assay for the quick and specific measurement of dabigatran and rivaroxaban in human plasma.

## Introduction

A new generation of oral anticoagulants known as direct thrombin inhibitors (DTI, dabigatran, etexilate) and the direct factor Xa inhibitors (DXaI, rivaroxaban, apixaban) have been approved for clinical use in patients with thrombosis prophylaxis in high-risk orthopedic patients and for stroke prevention in cases of non-valvular atrial fibrillation. In addition, the drugs are licensed for the treatment of and as secondary prophylaxis for deep vein thrombosis and pulmonary embolism, as well as—for rivaroxaban—a secondary prevention after acute coronary syndrome [1]. Further direct oral anticoagulants, such as the DXaI endoxaban, will be released soon [2]. Due to their pharmacological profiles, dabigatran, rivaroxaban and apixaban can be taken without routine monitoring [3–6]. On the other hand, assessing these drugs may be useful in emergency situations such as overdose, active bleeding, unknown medication, bridging with heparin or before surgery.

The influences on routine coagulation assays of direct oral anticoagulants (DOACs) have been described in several publications. For example, the effect on prothrombin time (PT) and activated partial thromboplastin time (aPTT) has been evaluated using various reagents, various applications and a wide range of laboratory instruments. Both PT and aPTT show a positive dose response to increasing DOAC concentrations; however, responsiveness varies based on the screening test and reagent [7–10].

Monitoring of the drugs can be done via clotting assays (diluted thrombin time, ecarin clotting time), chromogenic assays or liquid chromatography-mass spectrometry [11–15]. Whereas the functional assays show a good correlation between anti-Xa activity and apixaban/rivaroxaban plasma concentration or diluted thrombin time and dabigatran plasma concentration, there are some events (co-medication with low molecular weight heparin (LMWH) or unfractionated heparin (UFH), unknown medication) where measurement is not valid regarding DOACs. Recently, several LC-MS/MS assays have been described for the quantification of DOACs in plasma [13–15]. Each of these methods has its own advantage or disadvantage which will be discussed including the results of our LC-MRM MS method in the subsection “Comparison with other LC-MS assays” in the “Results and Discussion” section of this paper.

In the present study, a fast and sensitive UPLC-MRM MS method has been developed and validated for the simultaneous determination of dabigatran and rivaroxaban in human plasma. This method enables the user to measure the sample independent of co-medication, hemolysis or lipaemic/icteric plasma. Furthermore, a method comparison between the validated UPLC-MRM MS assay and the commercially available Direct Thrombin Inhibitor assay (DTI assay) for dabigatran measurement from CoaChrom Diagnostica and the anti-Xa assay for rivaroxaban measurement from Chromogenix were performed, respectively.

## Materials and Methods

### Reagents, internal standards, calibrators, and quality-control materials

Methanol and LC-MS-grade water were obtained from Fisher Scientific GmbH (Schwerte, Germany). Ammonium acetate, formic acid, and hydrochloric acid were purchased from Sigma-Aldrich (Deisenhofen, Germany). Dabigatran (the active form of dabigatran etexilate), [^13^C_6_]-dabigatran, rivaroxaban, and [^13^C_6_]-rivaroxaban were purchased from Alsachim (Strasbourg, France).

Primary stock solution of dabigatran, [^13^C_6_]-dabigatran, rivaroxaban, and [^13^C_6_]-rivaroxaban, each at a concentration of 10 mg/L, were prepared separately in methanol/water (50:50) and stored at -20°C. Using drug-free citrate plasma, we prepared several calibrators (0.8, 1.6, 3.1, 6.3, 12.5, 25.0, 50.0, 100, 200, 400 and 800 μg/L of both dabigatran and rivaroxaban) for the assay. Commercially available quality-control samples for dabigatran and rivaroxaban purchased from Technoclone GmbH (Vienna, Austria) were used.

An internal standard solution including 20 μg/L [^13^C_6_]-dabigatran, as well as 20 μg/L [^13^C_6_]-rivaroxaban, was prepared by mixing 1 ml [^13^C_6_]-dabigatran stock solution, 1 ml [^13^C_6_]-rivaroxaban stock solution and 498 ml methanol/water (90:10) containing 10 mmol/L hydrochloric acid.

### Plasma samples

Written informed consent was obtained from healthy volunteers who had not received any medication that could interfere with haemostasis during the week prior to blood sampling. All samples were collected within the Institute for Laboratory and Transfusion Medicine, Heart and Diabetes Center North Rhine-Westphalia, Ruhr University Bochum, Bad Oeynhausen, Germany. Furthermore, all samples were anonymized prior to inclusion in the study. Blood samples were taken using a 21-gauge butterfly needle with tubing and the corresponding tubes from Kabe^®^ (Primavette S^®^) (Nümbrecht-Elsenroth, Germany). The first 3–5 ml of blood was discarded. In order to obtain citrated blood for the different assays, blood was collected in 8.4 ml tubes containing 840 μl sodium citrate (100 mmol/L).

### Ethics statement

All plasma samples were collected in accordance with the German Act on Medical Devices (MPG, guideline 98/79/EG) for the collection of human residual material to evaluate suitability of an in vitro diagnostic medical device (§24). Hence, there was no need for an ethical approval as all materials used in this study were waste from routine laboratory diagnostics. Written informed consent was obtained from blood donors for the use of residual material of routine diagnostics for method development and quality assurance. The written informed consent was collected prior to the start of this research. The used plasma was residual material from voluntary healthy blood donors. The material was part of the sample collection for routine diagnostic; no collections specifically for the purpose of this study were performed. The voluntary blood donors received an expense allowance for the blood donation (see also §10 of the German Transfusion Act). None of the authors were directly involved in the plasma sample collection. No medical or personal data from the volunteers were collected for this study. All samples were anonymized before analysis.

### Sample preparation for UPLC-MRM MS analysis

Sample preparation was performed in a 1.5-ml polypropylene microcentrifuge tube. 100 μL each of citrate plasma sample, calibrator or quality-control sample were added to 900 μL internal standard solution (see above). The mixture was vortex-mixed for 5 s and after centrifugation at 14,000 x *g* at RT for 5 min, 500 μL of the clear, colorless supernatant was transferred to the autosampler vessel.

### UPLC-MRM MS analysis

For measurement of dabigatran and rivaroxaban, a 2.1 X 50-mm reverse phase column (Waters, Acquity UPLC BEH Phenyl, 1.7 μm) maintained at 50°C was used for separation by a UPLC system directly coupled to a Waters TQ tandem mass spectrometer (TQD) as described previously in details [16, 17]. A 1.0-μl sample was injected at a flow rate of 0.35 ml/min. The gradient program was as follows: Isocratic flow of 95%/5% water/methanol containing 0.1% formic acid and 2 mmol/L ammonium acetate was performed for 0.2 min, followed by a linear gradient over 1.5 min of 5%/95% water/methanol containing also 0.1% formic acid and 2 mmol/L ammonium acetate. After the isocratic elution of 95% methanol for 0.5 min, the mobile phase was reverted to the initial state. The run was terminated at 2.5 min. The TQD was operated in electrospray positive ionization mode. The system controls of the devices and data acquisition were performed using MassLynx NT 4.1 software. Data processing was performed by the MassLynx QuanLynx program which was provided with the instrument. Nitrogen was used as the nebulizing gas and Argon was used as the collision gas. Instrument settings were as follows: capillary voltage, 0.35 kV; source temperature, 105°C; desolvation temperature, 480°C. The collision gas pressure was 3.4 X 10^−3^ mbar. A sample analysis was performed in the multiple reaction monitoring mode (MRM) of the instrument. Sample cone voltage, collision energy, dwell time, and mass transitions for all compounds are listed in Table 1. The mass transition which was used for quantification of the DOACs (first transition) is written in bold type in Table 1.

**Table 1:** Multiple Reaction Monitoring (MRM) transitions monitored (m/z) with cone and collision energy.

Analyte	Transition	MRM (m/z)	Dwell (s)	Cone (V)	Collision Energy (eV)
**Dabigatran**	**first**	**472.2 > 289.2**	**0.05**	**38**	**27**
Dabigatran	second	472.2 > 306.2	0.05	38	20
Dabigatran	third	472.2 > 324.2	0.01	38	19
**[** ^**13**^ **C**_**6**_ **]-dabigatran**	**first**	**478.2 > 295.2**	**0.05**	**38**	**27**
[^13^C_6_]-dabigatran	second	478.2 > 312.2	0.05	38	20
[^13^C_6_]-dabigatran	third	478.2 > 330.2	0.01	38	19
**Rivaroxaban**	**first**	**436.1 > 145.0**	**0.05**	**40**	**29**
Rivaroxaban	second	436.1 > 231.2	0.05	40	21
Rivaroxaban	third	436.1 > 318.3	0.01	40	18
**[** ^**13**^ **C**_**6**_ **]-rivaroxaban**	**first**	**442.2 > 145.1**	**0.05**	**40**	**29**
[^13^C_6_]-rivaroxaban	second	442.2 > 237.2	0.05	40	21
[^13^C_6_]-rivaroxaban	third	442.2 > 324.0	0.01	40	18

### Ion enhancement and ion suppression effects

Ion enhancement and ion suppression effects were investigated by a post-column infusion experiment as described previously in details for mycophenolic acid [18].

### Validation

In accordance with our previously assay validation for nicotine and cotinine [16], mycophenolic acid and mycophenolic acid glucuronide [18], as well as the assay validation for six antiepileptic drugs [17], we used the STARD (Standard for Reporting of Diagnostic Accuracy) checklist [19, 20] and the report “Bioanalytical Method Validation–A Revisit with a Decade of Progress” [21] as the basis for validating the UPLC–MRM MS method for dabigatran and rivaroxaban to determine the most important test characteristics such as LOD, LLOQ, linearity, imprecision, and recovery.

### Linearity studies

A matrix-based calibration curve for both dabigatran and rivaroxaban was constructed using drug-free citrate plasma. 160 μL of a 10 mg/L dabigatran stock solution, as well as 160 μL of a rivaroxaban stock solution, were diluted with 1680 μL drug-free citrate plasma. The solution was mixed and used as calibrator 16. 1.0 ml of calibrator 16 was further diluted with 1 ml drug-free citrate plasma, mixed and used as calibrator 15. 1.0 ml of calibrator 15 was used to prepare calibrator 14 as described above, continuing this procedure until calibrator 1 was prepared. Plasma-based commercially available controls for both dabigatran and rivaroxaban were used.

### Limits of Detection (LOD) and Lower Limit of Quantification (LLOQ)

The minimum of detectable concentration was assessed as 3 SD_0_ added to the mean of the blank, where SD_0_ is the value of the standard deviation of the blank. The LOD was determined by performing 20 replicate measurements in a single UPLC-MRM MS assay with drug-free citrate plasma. For sensitivity determination, the lowest standard concentration in the calibration curve was considered as the LLOQ, provided that for this value precision was at least 20%.

### Precision

Intra-assay precision was determined by 20 replicate analysis of samples containing 26.8, 133.7, 264.6, 386.5, and 732.4 μg/L dabigatran, as well as 20 replicate analysis of samples containing 23.0, 113.7, 221.9, 423.9, and 850.1 μg/L rivaroxaban on the same day (see Table 2, concentrations A). Inter-assay precision was obtained by measurement of 20 replicate analysis of samples containing 26.7, 133.3, 260.0, 379.0, and 744.4 μg/L dabigatran, as well as 20 replicate analysis of samples containing 23.1, 111.4, 212.9, 408.8, and 827.9 μg/L rivaroxaban, but on 20 different days over the course of 1 month (see Table 2, concentrations B).

**Table 2:** Validation results of LOD, LLOQ, precision, recovery and accuracy.

Analyte	LOD	LLOQ	Level 1	Level 2	Level 3	Level 4	Level 5	^a^Ref. int.	^b^Recovery	^c^Acc. expected	^d^Acc. observed
Dabigatran ^e^Con. A	0.21	0.46	26.8	133.7	264.6	386.5	732.4	50.0–600.0	104.5	103.0	107.6 ± 1.5
Intraassay (CV, %)			3.3	2.0	2.4	1.3	1.2				
Dabigatran Con. B			26.7	133.3	260.0	379.0	744.4			280.0	286.7 ± 4.1
Interassay (CV, %)			5.7	2.9	2.0	1.8	1.3				
Rivaroxaban Con. A	0.34	0.54	23.0	113.7	221.9	423.9	850.1	50.0–600.0	87.0	60.0	62.4 ± 3.0
Intraassay (CV, %)			5.4	3.4	2.7	1.8	1.0				
Rivaroxaban Con. B			23.1	111.4	212.9	408.8	827.9			305.3	307.8 ± 3.7
Interassay (CV, %)			8.4	4.6	4.1	4.6	2.5				

^a^Ref. Int., Reference interval (μg/L);

^b^Recovery (%) was performed in the Ref. int.; Recovery range for dabigatran and rivaroxaban were 83.8%– 113.0% and 73.6%– 105.4%, respectively.

^c^Acc. expected = Accuracy expected;

^d^Acc. observed = Accuracy observed (mean ± SD); Measurements were performed using quality control samples.

^e^Con. = Concentration (μg/L)

### Stability

The stability of dabigatran and rivaroxaban in citrate plasma was investigated by measuring these compounds in a low, medium, and highly concentrated samples stored at -20°C, 4°C, and RT after 1 day, 1 week and 1 month, respectively.

### Recovery

The recovery efficiency of the assay was established by measuring the concentration of both dabigatran and rivaroxaban in citrate plasma before and after enrichment with different amounts of dabigatran and rivaroxaban, respectively. Analytical recoveries were calculated as the measured concentrations divided by the expected concentrations and expressed as a percentage.

### Method comparison

Plasma samples from at least 6 different healthy individuals were used to prepare 55 plasma samples which then were spiked with different concentrations of dabigatran and rivaroxaban, respectively. The UPLC-MRM MS method proposed here was compared with the commercially available automated dabigatran assay from CoaChrom Diagnostica (Maria Enzersdorf, Austria), as well as rivaroxaban anti-Xa assay from Chromogenix (Orangeburg, NY, USA), by measuring the same citrate plasma samples spiked with different amount of dabigatran and rivaroxaban, respectively. Statistical analyses of the results were done using MedCalc Version 11.6.1.0.

### Coagulation analysis

The coagulation measurements were performed automatically on an ACL TOP 700 system from Instrumentation Laboratory (Kirchheim, Germany). For the prothrombin time, we used RecombiPlasTin 2G and for partial thromboplastin time SynthASil reagent, both from Instrumentation Laboratory (Kirchheim, Germany). All laboratory tests were performed automatically at the control temperature of 37°C and the clotting formation was measured by a turbidimetric method for dabigatran determination and the color development was measured at 405 nm for rivaroxaban determination, respectively. For calibration and control commercially available calibrators for dabigatran and rivaroxaban, as well as quality-control samples for both drugs from Technoclone GmbH (Vienna, Austria) were used.

## Results and Discussion

### General approaches of the UPLC-MRM MS method

**Fig 1:**
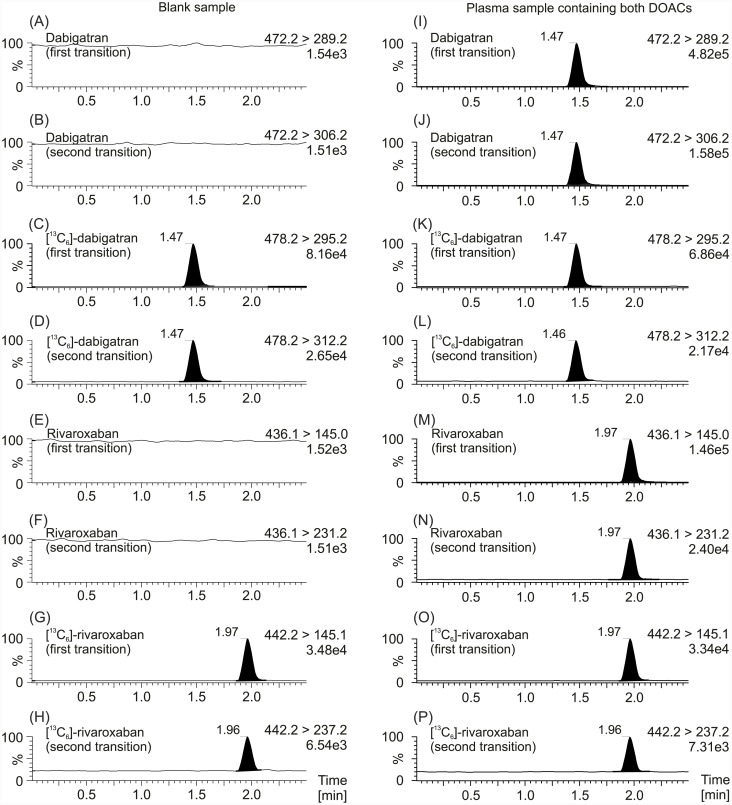
MRM chromatograms of plasma samples with and without DOACs. MRM chromatograms of a plasma sample without any DOACs are depicted on the left (A, B, E, F), as well as MRM chromatograms of a plasma sample containing both dabigatran and rivaroxaban are depicted on the right (I, J, M, N). MRM chromatograms of the internal standard [^13^C_6_]-dabigatran (C, D, K, L) concerning dabigatran, as well as the internal standard [^13^C_6_]-rivaroxaban (G, H, O, P) concerning rivaroxaban are also shown. The first mass transition which was used for quantification of the drug, as well as the second mass transition which was used for verification is plotted. Data were normalized to largest peak in the plots. An estimated peak height is shown in the plots below the transition remark.

**Fig 2:**
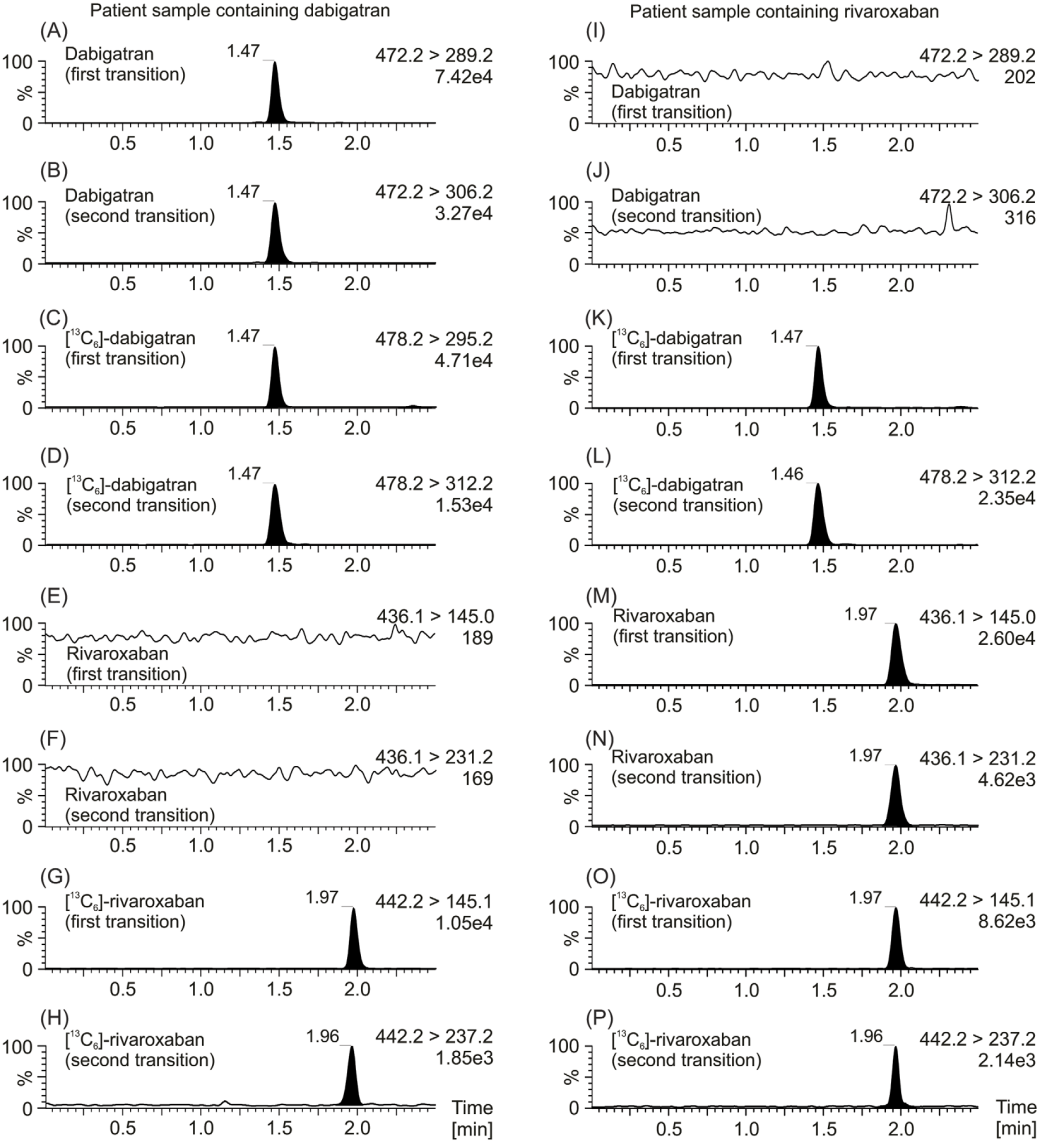
MRM chromatograms of patients’ plasma samples. MRM chromatograms of a plasma sample from a patient which have been treated with dabigatran are depicted on the left (A, B, E, F), as well as MRM chromatograms a plasma sample from a patient which have been treated with rivaroxaban are depicted on the right (I, J, M, N). MRM chromatograms of the internal standard [^13^C_6_]-dabigatran (C, D, K, L) concerning dabigatran, as well as the internal standard [^13^C_6_]-rivaroxaban (G, H, O, P) concerning rivaroxaban are also shown. The first mass transition which was used for quantification of the drug, as well as the second mass transition which was used for verification is plotted. Data were normalized to largest peak in the plots. An estimated peak height is shown in the plots below the transition remark.

**Fig 3:**
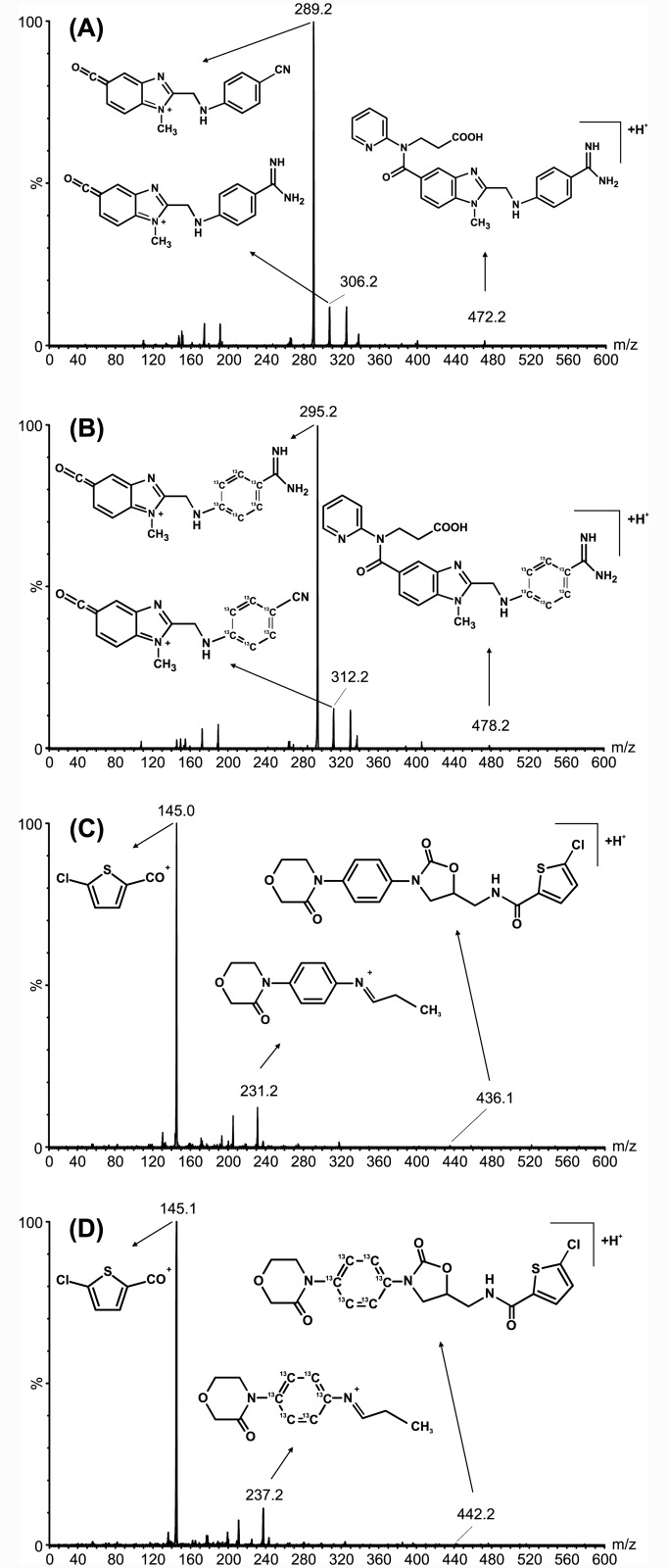
Product ion spectra of DOACs. Product ion spectra of dabigatran (A) and rivaroxaban (C), as well as its internal standards [^13^C_6_]-dabigatran (B) and [^13^C_6_]-rivaroxaban (D) are depicted. The MH^+^ precursor ions and the fragment ions are shown. The chemical structures of the molecules are depicted. In addition, the positions of the ^13^C_6_ atoms of the internal standards [^13^C_6_]-dabigatran (B) and [^13^C_6_]-rivaroxaban (D) are shown.

Sample preparation by means of a simple protein precipitation procedure using IS precipitation solution produced, after a short centrifugation step, a clear supernatant that gave an interference-free chromatogram for all compounds (Figs 1 and 2). Just as described in the previous LC-MS/MS methods for dabigatran and rivaroxaban measurements we used [^13^C_6_]-dabigatran and [^13^C_6_]-rivaroxaban as the most appropriate IS due to their similar structure and their lack of clinical use [13–15]. Systematic optimization of LC-MRM MS measurements shows that positive mode yielded a better mass spectrometer response than the negative mode. The most sensitive mass transitions of the two DOACs, as well as its ISs were used for determination of the drugs (Table 1). Chromatographic conditions were optimized though several trials in order to achieve suitable sensitivity, as well as short run time. All compounds were clearly separated from the void volume (retention time <0.3 min) and elute in <2.0 min, permitting an injection-to-injection cycle time of 2.5 min which allows a faster measurement of dabigatran and rivaroxaban than with the previous LC-MS/MS methods [13–15]. The most sensitive measurement for dabigatran and rivaroxaban, as well as its corresponding [^13^C_6_]-isotopes, were achieved by monitoring the fragmentation of single-charged molecule ions (dabigatran + H^+^), ([^13^C_6_]-dabigatran + H^+^), (rivaroxaban + H^+^), and ([^13^C_6_]-rivaroxaban + H^+^) with m/z transitions of m/z 472.2 → 289.2, m/z 478.2 → 295.2, m/z 436.1 → 145.0, and m/z 442.2 → 145.1, respectively, whereas the conformation ratio was determined from the ratio m/z 472.2 → 306.2 (dabigatran + H^+^), m/z 478.2 → 312.2 ([^13^C_6_]-dabigatran + H^+^), 436.1 → 231.2 (rivaroxaban + H^+^), and 442.2 → 237.2 ([^13^C_6_]-rivaroxaban + H^+^). In addition, a third transition for more comprehensive identification was added (see Table 1). As a compromise between sensitivity and good characterization of peak shapes about 8 mess points per peak were recorded. Contrary to the previous LC-MS/MS method for measurement of DOACs our UPLC-MRM MS method use a first transition for quantification of the drugs, as well as for its ISs and in addition, a second transition for qualification to detect interferences which may be present in complex biological matrix such as human plasma and which could interfered with measurement accuracy and, furthermore, a third transition for more comprehensive identification [13–15]. Overall, the use of two transitions per substance leads to increase in specificity of the method. The dabigatran fragment ion of m/z 289.2 was formed from the single-charged dabigatran ion (m/z 472.2) via m/z 324.2 by means of an intramolecular six-ring cyclic rearrangement reaction with loss of ketene (CH_2_CO) and 2-(methylenimino)pyridine (C_6_H_6_N_2_) and subsequently via m/z 307.2 and 306.2 through loss of water and ammonia, respectively [22]. Concerning the two major fragments of m/z 231.2 and 145.1 from the single-charged rivaroxaban ion (m/z 436.1), it was suggested that these fragments were obtained by cleavage of the oxazolidinone and the chlorothiophene amide moiety, respectively [23]. We generated product ion spectra of both dabigatran and rivaroxaban which are shown in Fig 3.

### Ion suppression and ion enhancement effects

**Fig 4:**
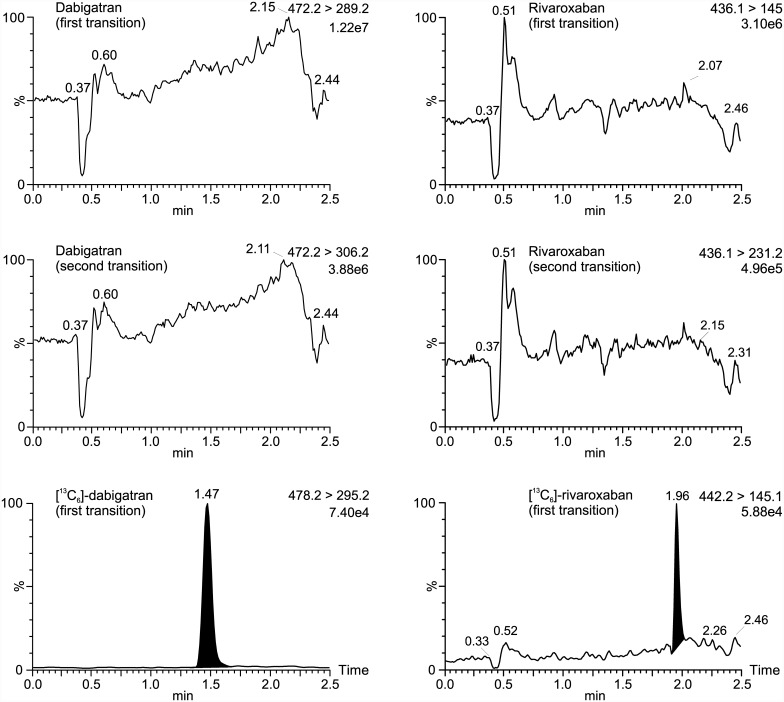
Ion suppression profiles of DOACs. Ion suppression profile for dabigatran is depicted on the left and for rivaroxaban is depicted on the right, respectively, performed with a post-column flow injection of 0.1 mg/L drug into the UPLC elute of drug-free samples. In addition, the mass transition of the corresponding internal standard is shown. Data were normalized to largest peak in the plots. An estimated peak height is shown in the plots below the transition remark.

Ion suppression and ion enhancement effects attributable to the sample matrix were investigated as described elsewhere for mycophenolic acid [18]. A typical ion chromatogram in which the response of the MRM transition of dabigatran, as well as rivaroxaban, was continuously monitored as shown in Fig 4. Most ion suppressions or enhancements were observed at 0.3 to 1.0 min before and at 2.2 to 2.5 min after the [^13^C_6_]-dabigatran signal (retention time, 1.47 min) and [^13^C_6_]-rivaroxaban signal (retention time, 1.97 min). Therefore, no significant ion suppressions influenced the analytes signals accordingly; there was no loss of sensitivity of the measurement. Furthermore, quantitative errors resulting from potential ion suppression are compensated via the internal standards ([^13^C_6_]-isotope), which are structurally identical to the corresponding analyte and eluted at the same retention time.

### Validation

Carryover from the 280/156 μg/L dabigatran/rivaroxaban control to the drug-free citrate sample was less than 0.1% for both drugs. The calibration curve of both dabigatran and rivaroxaban were linear over the working range between 0.8 and 800 μg/L (r >0.99). This field covers typical expected plasma concentrations of dabigatran (e.g. for stroke prevention) which was under steady-state conditions about 175 μg/L (117–275 μg/L, 25th-75th percentile range), measured around 2 hours after 150 mg dabigatran etexilate administration twice daily (specialized information, Pradaxa, Boehringer Ingelheim, Germany), as well as expected plasma concentrations of rivaroxaban (e.g. for treatment of acute deep vein thrombosis) with a concentration of about 215 μg/L (22–535 μg/L, 90% prediction interval), measured around 2–4 hours after 20 mg rivaroxaban once daily (specialized information, Xarelto, Bayer Pharma AG, Germany). The limit of detection (LOD) was < 0.4 μg/L, whereas the limit of quantification (LLOQ) was < 0.6 μg/L for both anticoagulants in the LC-MRM MS assay, which is clearly lower than the typical plasma concentration of dabigatran- or rivaroxaban-treated patients. LOD and LLOQ of our LC-MRM MS method were slightly lower in comparison to the existing LC-MS/MS methods for DOAC measurement which were about 1 μg/L or higher (see Table 3) [13–15, 24], implying that our method was a little bit more sensitive (see subsection “Comparison with other LC-MS assays”). Accuracy, recovery, intra-assay and inter-assay precision for dabigatran and rivaroxaban were high, as presented in Table 2. These assay parameters of our method are broadly comparable with the existing LC-MS/MS methods for DOACs [13–15].

**Table 3:** LC-MS/MS method comparison for the determination of DOACs.

DOAC	Matrix	LC type	Sample clean-up	MS type (number of transitions)	AnalysisTime (min)	^a^LOD (μg/L)	^b^LLOQ (μg/L)	^c^Pre-cision (% CV)	Accu-racy (%)	Linearity Range (μg/L)	^d^Ref.
Apixaban	human plasma / blood	BEH C8	^e^PP (^f^MeCN)	^g^MRM (1)	5.0	≤ 0.025	No data	7.5–9.5	91–95	23–750	13
Dabigatran	human plasma / blood	BEH C8	PP (MeCN)	MRM (1)	5.0	≤ 0.025	No data	< 2.8	101–106	23–750	13
Rivaroxaban	human plasma / blood	BEH C8	PP (MeCN)	MRM (1)	5.0	≤ 0.025	No data	4.2–7.8	114–116	23–750	13
Apixaban	human plasma	PhenylHexyl	PP (MeCN) + ^h^TFC	^i^FSM+MS^2^	6.0	No data	1.0	2.6–4.3	92–101	1.0–500	14
Dabigatran	human plasma	PhenylHexyl	PP (^j^HCA) + TFC	FSM+MS^2^	6.0	No data	1.0	5.0–6.6	98–105	1.0–500	14
Edoxaban	human plasma	PhenylHexyl	PP (MeCN) + TFC	FSM+MS^2^	6.0	No data	1.0	2.9–7.9	97–104	1.0–500	14
Rivaroxaban	human plasma	PhenylHexyl	PP (MeCN) + TFC	FSM+MS^2^	6.0	No data	1.0	3.1–7.1	98–102	1.0–500	14
Dabigatran	human plasma / blood	BEH C18	PP (methanol/HCl)	MRM (1)	^k^~ 4.0	No data	2.5	2.9–9.4	96–98	2.5–500	15
Rivaroxaban	human plasma / blood	BEH C18	PP (methanol/HCl)	MRM (1)	~ 4.0	No data	2.5	2.5–6.6	97–104	2.5–500	15
Dabigatran	human plasma	BEH C8	PP (methanol/HCl)	MRM (2)	4.5	1.0	2.0	4.8–11.9	94–109	1.0–500	24
Dabigatran	rate plasma	XB-C18	PP (methanol/HCl)	^l^SRM (1)	7.0	No data	1.0	7.6–9.1	99–104	1.0–500	29
Apixaban	human plasma	C18	PP (MeCN)	SRM (2)	2.5	0.09	0.3	0.7–3.7	110–111	1.0–500	30
Dabigatran	human plasma	C18	PP (MeCN)	SRM (2)	2.5	0.07	0.2	2.7–11.8	91–101	1.0–500	30
Rivaroxaban	human plasma	C18	PP (MeCN)	SRM (2)	2.5	0.30	0.9	1.4–5.5	104–106	1.0–500	30
Dabigatran	human plasma	BEH Phenyl	PP (methanol/HCl)	MRM (3)	2.5	0.21	0.5	1.3–5.7	102–105	0.8–800	This paper
Rivaroxaban	human plasma	BEH Phenyl	PP (methanol/HCl)	MRM (3)	2.5	0.34	0.5	2.5–8.4	101–104	0.8–800	This paper

^a^LOD: Limit Of Detection;

^b^LLOD: Lower Limit Of Quantification;

^c^Precision: Inter-day precision;

^d^Ref.: References;

^e^PP: protein precipitation;

^f^MeCN: acetonitrile;

^g^MRM: multiple reaction monitoring;

^h^TFC: TurboFlow column (Cyclone C18-P-XL) [clean-up];

^i^FSM+MS^2^: full scan mode with single data-dependent fragmentation (MS^2^) scans;

^j^HCA: 0.1 mol/L aqueous hydrochloric acid;

^k^~: nearly;

^l^SRM: selected reaction monitoring. The sample volume was approximately 100 μl for all methods.

Dabigatran and rivaroxaban were stable in citrate plasma for at least 1 week at -20°C (after thawing), 4°C, RT, and 37°C, which was ascertained with systematic testing over a period of 1 month. Both compounds seem to be very stable in plasma. Our results corresponds with previous studies which present that both DOACs showed adequate stability during three freeze–thaw cycles, 24 h of plasma storage at room temperature and 4°C, and 72 days of plasma storage at a temperature lower than -80°C [13].

### Method comparison with commercially available coagulation-based assays

**Fig 5:**
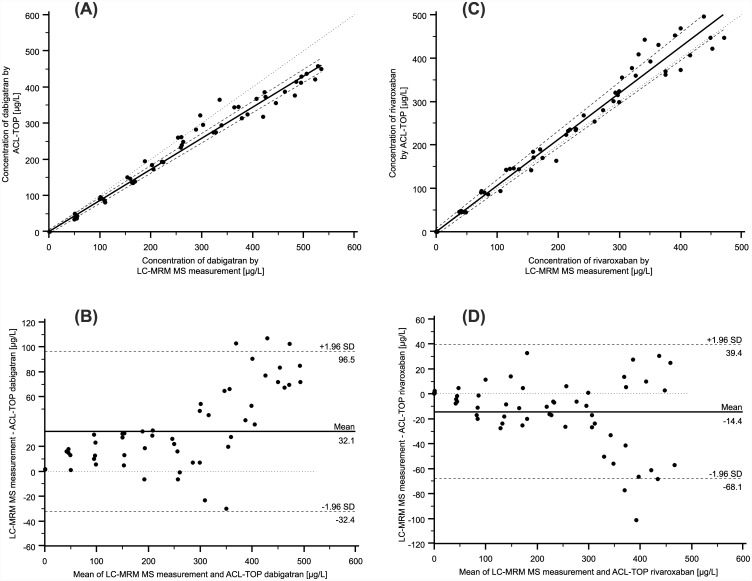
Method comparison using Passing-Bablok regression and Bland-Altman plot. (A) Comparison of dabigatran results obtained by the LC-MRM MS assay and the DTI assay used for dabigatran measurement from CoaChrom Diagnostica performed on the ACL-TOP analyzer by Passing-Bablok regression: DTI assay = 0.86 (LC-MRM MS)– 1.03 [μg/L] (r = 0.99; n = 55; 95% CI for slope, 0.84–0.89; 95% CI for intercept, -6.10 μg/L—4.35 μg/L). (B) Bland-Altman plot for the comparison of LC-MRM MS assay vs. DTI assay from CoaChrom Diagnostica. The mean value (n = 55) of the two methods is plotted against the difference between the two values (LC-MRM MS assay–DTI assay from CoaChrom Diagnostica). The mean difference between the two methods was 32.1 μg/L. The mean (–) and ± 2 SD lines (---) are plotted for references. (C) Comparison of rivaroxaban results obtained by LC-MRM MS assay and the anti-Xa assay used for rivaroxaban from Chromogenix performed on the ACL-TOP analyzer by Passing-Bablok regression: anti-Xa assay = 1.07 (LC-MRM MS) + 0.00 [μg/L] (r = 0.98; n = 55; 95% CI for slope, 1.00–1.12; 95% CI for intercept, -7.10 μg/L– 7.80 μg/L). (D) Bland-Altman plot for the comparison of LC-MRM MS assay vs. anti-Xa assay used for rivaroxaban from Chromogenix. The mean value (n = 55) of the two methods is plotted against the difference between the two values (LC-MRM MS assay–anti-Xa assay from Chromogenix). The mean difference between the two methods was -14.4 μg/L. The mean (–) and ± 2 SD lines (---) are plotted for references.

Method comparison analysis between our UPLC-MRM MS method and the commercially available automated DTI assay used for dabigatran measurement from CoaChrom Diagnostica and the commercially available automated anti-Xa assay used for rivaroxaban measurement from Chromogenix, respectively, was performed using 55 samples of drug-free citrate plasma spiked with dabigatran, as well as a further 55 samples of drug-free citrate plasma spiked with rivaroxaban. Both commercially available assays were performed on the ACL-TOP analyzer. As shown in Fig 5A and 5C, both method comparisons showed a high degree of correlation. The Passing-Bablok regression analysis revealed an intercept of -1.03 μg/L (95% confidential interval (95% CI), -6.10 to 4.35 μg/L) and a slope of 0.86 μg/L (95% CI, 0.84 to 0.89 μg/L) for dabigatran (Fig 5A), and an intercept of 0.00 μg/L (95% CI, -7.10 to 7.80 μg/L) and a slope of 1.07 μg/L (95% CI, 1.00 to 1.12 μg/L) for rivaroxaban (Fig 5C). The correlations coefficient was 0.988 for dabigatran and 0.984 for rivaroxaban. In order to further assess the agreement between the two measurement techniques, the difference between the results of the two methods was plotted against the average of the two methods as described by Bland and Altman [25, 26]. The Bland and Altman plot of dabigatran measurements showed a proportional error in cases where high concentrations were measured (Fig 5B), and the Bland and Altman plot of rivaroxaban results showed that the variation of at least one method depends on the magnitude of measurements (Fig 5D).

**Fig 6:**
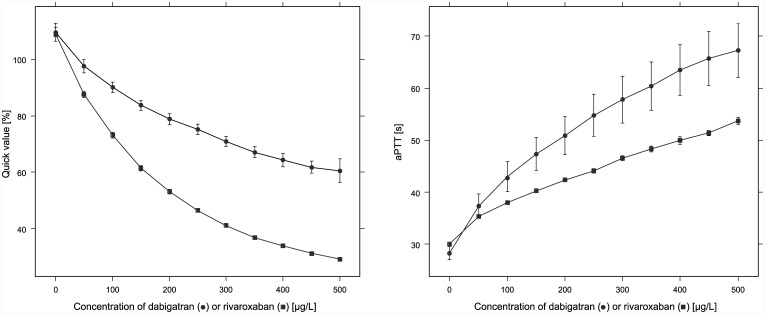
Effects of dabigatran and rivaroxaban on blood clotting. Quick values and aPTT of dabigatran or rivaroxaban spiked blood samples. The mean and standard deviation of two measurements of each case are depicted.

**Fig 7:**
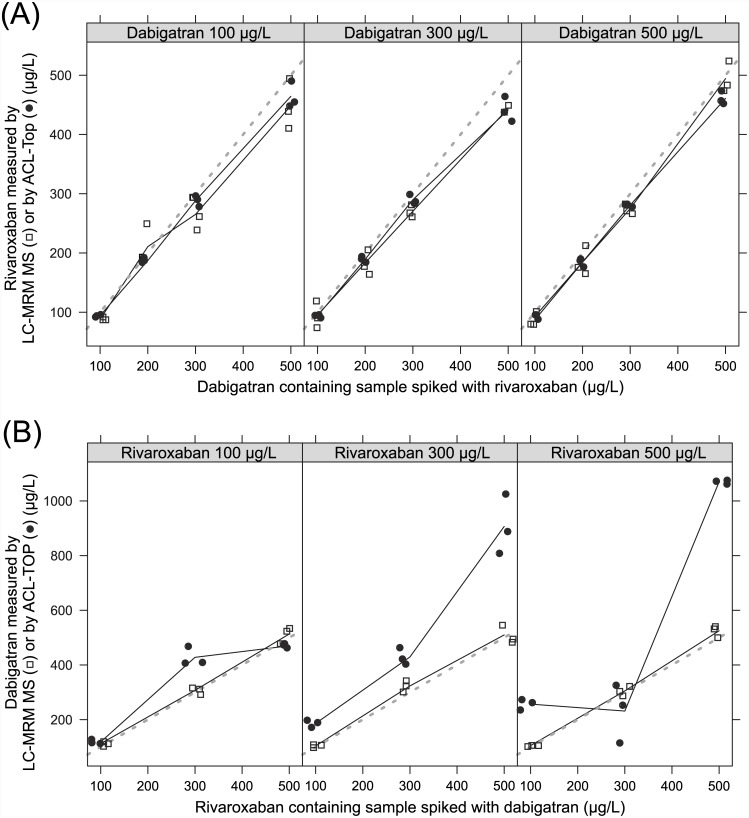
Mutual influences of DOAC measurement. (A) Measurement of rivaroxaban in rivaroxaban and dabigatran spiked plasma. Concentrations were quantified using our UPLC-MRM MS assay (free squares), as well as the automated anti-Xa assay used for rivaroxaban measurement from Chromogenix on the ACL-TOP analyzer (filled circles). For clarification, the measuring points were connected (solid line). The line of identity X = Y is also shown (dotted line). (B) Measurement of dabigatran in rivaroxaban and dabigatran spiked plasma. Concentrations were quantified using our UPLC-MRM MS assay (free squares), as well as the automated DTI assay for dabigatran measurement from CoaChrom Diagnostica performed on the ACL-TOP analyzer. For clarification, the measuring points were connected (solid line). The line of identity X = Y is also shown (dotted line).

Furthermore, we investigated the effects of dabigatran and rivaroxaban using global coagulation assays by measurement of aPTT and PT, whereby the Quick value was calculated using the PT. Therefore, we used on the one hand dabigatran and on the other hand rivaroxaban spiked plasma samples. Increasing concentrations of dabigatran or rivaroxaban showed an expected elevation of aPTT, as well as PT which corresponds to a lower Quick value, as shown in Fig 6. Our results are consistent with the reported data described in the literature [8, 11, 12, 27]. In a further experiment, we measured one of the drugs in samples which were spiked with both of them. As shown in Fig 7A different dabigatran concentrations in the samples have nearly no influence on the rivaroxaban measurements regardless of whether the measurement was performed by mass spectrometry or by the automated anti-Xa assay used for rivaroxaban measurement from Chromogenix on the ACL-TOP analyzer. The measured values of both methods displayed good correlation with the spiked concentration of rivaroxaban in the samples with low, medium, and high dabigatran concentration, respectively. In contrast, determination of dabigatran in samples spiked with rivaroxaban showed good correlation by mass spectrometry measurement regarding increasing concentrations of dabigatran in samples with low, medium, and high rivaroxaban concentrations, respectively. Poor correlation was found for automated DTI assay used for dabigatran measurement from CoaChrom Diagnostica performed on the ACL-TOP analyzer (Fig 7B). Especially in samples with high rivaroxaban concentrations the correlation with increasing dabigatran quantity was very poor probably because high rivaroxaban concentrations interfere with the DTI assay. No suitable data were obtained using the diluted thrombin time. Most of the samples measured by this application showed values higher than expected (Fig 7B). One possible explanation could be that the added rivaroxaban reduces the amount of endogenous thrombin. Overall, one can say that the measurement of dabigatran in the presence of rivaroxaban, as well as the measurement of rivaroxaban in the presence of dabigatran is unproblematic using our mass spectrometric method in contrast to certain other measurement techniques for these drugs such as classical clotting or chromogenic assays.

In routine laboratory diagnostics, a further advantage/benefit of mass spectrometry analysis of the drug rivaroxaban in the clinical setting could be overlapping therapy of preoperative discontinuation of DOAC and bridging with low molecular weight heparin (LMWH), although such a therapy is controversially discussed [28]. In this case it is not possible to measure the residual amounts of rivaroxaban using a standard coagulation assay because both anticoagulants (LMWH and rivaroxaban) influence the coagulation.

### Comparison with other LC-MS assays

First LC-MS methods for quantification of DOACs were recently published [13–15, 24, 29, 30]. However, these methods differ in some cases significantly from our method. The most important differences between our method and previously published DOAC assays are shown in Table 3. As can be seen in this table, our UPLC-MRM MS method has an analysis time of 2.5 min which turns out to be one of the fastest together with the assay reported by Blaich et al. [30]. The precision for dabigatran measurement of our method was higher than the precision of the other methods with the exception of the method reported by Schmitz et al. [13] which shows the highest precision for this DOAC. In addition, our method shows a wide linear measuring range, larger than the previously published DOAC methods for dabigatran and rivaroxaban, respectively. Most previously published LC-MS/MS methods for DOACs measurement used only one mass transition for quantification [13, 15, 29], two methods used a further second mass transition for qualification [24, 30], one method measured the DOACs by full scan mode with single data-dependent fragmentation (MS^2^) scans, whereas our UPLC-MRM MS method used a mass transition for quantification, a further mass transition for qualification, and an additional third mass transition for more comprehensive identification. LOD, LLOQ, and accuracy of our method were comparably well for both compounds according to previously published DOAC LC-MS assays (Table 3). Furthermore, Table 3 shows also that no significant differences in response could be seen between samples prepared from plasma and those prepared from whole blood, indicating that the studied DOACs are not adsorbed to erythrocytes [13].

### LC-MS measurement of DOACs as routine clinical diagnostic application

LC-MS has been increasingly used in routine clinical laboratories and more and more clinical parameters including amino acids, proteins, peptides, lipids, and lipoproteins were measured by this technique [31–33]. In addition, MS-based clinical assays which provide rapid and multicomponent analysis with high sensitivity and selectivity are used more often for drug monitoring and toxicological examinations [16–18, 34, 35]. Last but not least there are also LC-MS methods developed which were used to measure enzyme activities [36–39]. The high specificity, sensitivity, and the possibility to measure several analytes simultaneously make it an ideal alternative to immunoassays. Furthermore, LC-MS offers higher flexibility than immunoassays and abundant information can be received from a single LC-MS run [31]. However, several limitations of LC-MS obviously exist such as high instrument cost, the need of expertise, and the need of time-consuming validation of the LC-MS assay. Maybe these limitations can be overcome in the future with easier to handle as well as low-cost equipment. Our fast, precise, and direct measurement of DOACs by LC-MS in patients’ plasma is a further MS-based application suitable for routine measurement of dabigatran and rivaroxaban which gives information about the drug level in patients’ blood and is therefore helpful in different clinical circumstances such as in the case of suspicion of overdose, when patients switch from existing oral anticoagulant, in patients with hepatic or renal impairment, by concomitant use of interaction drugs, or to assess anticoagulant concentration in patients’ blood before major surgery.

## Conclusions

A specific, sensitive and very fast ultra-performance liquid chromatography electrospray-tandem mass spectrometry assay using stable isotope standards was developed and validated for the simultaneous determination of rivaroxaban and dabigatran in human plasma. The method was successfully applied to determine the concentrations of the two drugs independent of interference factors such as hemolysis or lipaemic plasma. This study additionally compares the performance of the two different analytical methods for determining concentrations of rivaroxaban and dabigatran in plasma samples showing a high correlation between standard diagnostic assays and the newly developed mass spectrometry method. The newly developed method may be useful in risk assessment in patients undergoing DOACs treatment.
